# Exploratory investigation of urinary alkanes and other volatile organic compounds in paediatric patients with tuberculous meningitis

**DOI:** 10.1007/s11306-025-02304-5

**Published:** 2025-08-11

**Authors:** Simon Isaiah, Du Toit Loots, A. Marceline Tutu van Furth, Regan Solomons, Sabine van Elsland, Martijn van der Kuip, Shayne Mason

**Affiliations:** 1 Human Metabolomics, Faculty of Natural and Agricultural Sciences, North-West University, Potchefstroom, South Africa; 2 Pediatric Infectious Diseases and Immunology, Amsterdam University Medical Centers, Emma Children’s Hospital, Vrije Universiteit, De Boelelaan 1117, Amsterdam, The Netherlands; 3 Department of Paediatrics and Child Health, Faculty of Medicine and Health Sciences, Stellenbosch University, Cape Town, South Africa; 4 MRC Centre for Global Infectious Disease Analysis, School of Public Health, Imperial College London, London, UK

**Keywords:** Urine, Alkanes, Volatile organic compounds (VOCs), Paediatric, Tuberculous meningitis (TBM), Metabolomics, Gas chromatography coupled with time-of-flight mass spectrometry (GC-TOFMS)

## Abstract

**Background:**

Tuberculous meningitis (TBM) is a disease caused by *Mycobacterium tuberculosis* (*M. tb*) infection of the brain. Alkanes and other volatile organic compounds (VOCs) are biologically important metabolites that are used by infectious mycobacteria species for growth and survival strategies.

**Objective:**

This study investigated the altered alkanes and other VOCs in the urine from paediatric cases with TBM.

**Method:**

We used untargeted gas chromatography coupled with time-of-flight mass spectrometry (GC-TOFMS) to analyse and compare all volatile, underivatised compounds present in the urine from 27 confirmed cases of paediatric TBM over a treatment period of six months, as well as a control group (*n* = 13).

**Result:**

Four elevated alkanes (pentadecane, 5,7-dimethyl-undecane, 4,7-dimethyl-undecane, and 2,6-dimethyl-undecane), three alkenes (decreased 2,5-dimethyl-2-hexene and 4,4-dimethyl-1-pentene, and increased 3-methoxy-1-pentene), and three other VOCs of biological interest (decreased 2-butenoic acid methyl ester and 3-heptanone, and increased 2-pyrrolidinone) were identified as statistically significant. These volatile compounds remained perturbed during the TBM treatment.

**Conclusion:**

This study discovered new systemic metabolic information about *M. tb* in the host and the role of alkanes and VOCs in the potential persistence of *M. tb*. We demonstrate the value of targeting alkanes and other VOCs for future metabolomics studies of *M. tb*.

**Supplementary Information:**

The online version contains supplementary material available at 10.1007/s11306-025-02304-5.

## Introduction

Tuberculous meningitis [TBM; tuberculosis (TB) of the brain] is the most severe extrapulmonary form of TB, and if not diagnosed early, can result in a high probability of death or long-term neurological complications. Definite TBM is diagnosed based on microbiological evidence of *Mycobacterium tuberculosis* (*M. tb*) in the cerebrospinal fluid (CSF). However, the paucity of *M. tb* in the CSF can sometimes lead to an inconclusive differential diagnosis of TBM. Hence, there is a desperate need to discover new potential diagnostic markers to aid in quicker and more efficient diagnosis of TBM.

Alkanes are saturated hydrocarbons, meaning that they consist of a series of single-bonded carbon and hydrogen atoms. It is known from the literature that many species of bacteria use alkanes as a carbon source (Rojo, 2009; Wentzel et al., 2007). Although sparse in the literature, some studies have shown that species of *Mycobacterium* use alkanes as a carbon source. Dunlap and Perry showed that C13-C17 alkanes were incorporated into the fatty acid metabolism of an OFS lab strain of a *Mycobacterium* species (Dunlap & Perry, 1967); however, alkanes shorter than C13 or longer than C17 were not incorporated into the cellular fatty acids of mycobacteria without some form of degradation (Dunlap & Perry, 1968). Churchill et al. (1999) isolated a CH1 lab strain of a *Mycobacterium* species and showed that mycobacteria could use a wide range of alkanes [linear (dodecane and hexadecane); branched-chain (pristane); long-chain (octadecane, docosane, and octacosane)] as the sources of carbon and energy. Another study by Van Beilen et al. (2002) showed that various species of mycobacteria could use C6-C24 alkanes as carbon sources. To use alkanes, bacteria need the alkane hydroxylase system, which consists of three protein components: alkane hydroxylase (AlkB), rubredoxin (AlkG), and rubredoxin reductase (AlkT) (Smits et al., 2002). Stokas et al. (2022) showed that *Mycobacterium tuberculosis* (*M. tb*), the pathogen responsible for tuberculosis (TB), regulates a highly conserved alkane hydroxylase/rubredoxin system that contributes to the success of these specific mycobacteria in human macrophages. Hence, *M. tb* uses alkanes for growth and survival.

The breath of active cases of pulmonary TB has been investigated (Phillips et al., 2007, 2010), and it was found that the alkanes tridecane, 4-methyldodecane, and 3,7-dimethyldecane were among the volatile organic compounds (VOCs) in the breath that were diagnostic markers of TB. Additionally, urinary VOCs have been shown to have diagnostic potential by differentiating TB patients from controls (Banday et al., 2011; Lim et al., 2016). However, alkanes and VOCs have never been targeted in any TBM study nor investigated in other biofluids (urine, plasma, serum, etc.) from TBM cases. VOCs in biofluids are low-molecular-weight compounds that are easily vaporised when heated. Here, we conducted an exploratory investigation of alkanes and other VOCs in urine collected from paediatric cases undergoing treatment for confirmed TBM. For this study, we used gas chromatography coupled with time-of-flight mass spectrometry (GC-TOFMS) as the analytical approach, without derivatising the samples (derivatisation would result in loss of VOCs). Hence, the aim of this study was to evaluate the urinary profile of alkanes and other VOCs in cases of TBM.

## Materials and methods

### Sampling and ethics

This investigation involved retrospectively collected urine samples (van Elsland et al., 2018) from infants and children (aged < 13 years) residing in the Western Cape Province of South Africa, a geographical location known for its high prevalence of TB (681 per 100,000), especially among children (Donald et al., 1996; Mandalakas et al., 2021; van Toorn & Solomons, 2014). Children exhibiting clinical symptoms and signs indicative of meningitis, initially seen at the basic level and regional health facilities, were referred to the Department of Paediatrics and Child Health at Tygerberg Hospital in the Western Cape Province of South Africa. Following the application of the universal research case definition criteria for TBM (Marais et al., 2010), all TBM participants in our cohort (n = 27) were diagnosed with definite TBM. The diagnosis of definite TBM was based on the detection of *M. tb* in CSF by microscopy, culture, and/or a commercial nucleic acid amplification test. All TBM patients were stabilised in the Tygerberg Hospital Paediatric Neurology ward and treated with an intensified anti-tuberculous drug regimen comprising high-dose rifampicin, isoniazid, pyrazinamide, and ethionamide (Van Toorn et al., 2012). Urine samples were collected with the consent of the parents or guardians and the assent of the child if older than 7 years and can comprehend. All experimental samples were collected at baseline (time 0; T0 – collected upon discharge from the hospital; the median time from admission to discharge in the study setting over the past 38 years was 16 days, the interquartile range was 12–23 days) and at one-month intervals at the patient out-clinic, up to six months (T1-T6), until the completion of the TBM treatment (van Elsland et al., 2018). The control group (n = 13) consisted of anonymous paediatric patients with written and informed parental or guardian consent and the child’s assent, if older than 7 years, who were negative for meningitis, without neurological symptoms, and from the same geographic region as the TBM patients; classified as ‘normal and healthy cases’. Ethical approval was granted by the Stellenbosch University Health Research Ethics Committee (HREC) (ethics approval number: N16/11/142), the Western Cape Department of Health and Wellness, and the HREC of North-West University, Potchefstroom campus (ethics approval number: NWU-00063-18-A1-01). The presence of HIV co-infection further complicates an already intricate metabolic profile; hence, individuals with positive or unknown HIV status were excluded from this study.

### Sample handling, storage, and transportation

**Fig. 1:**
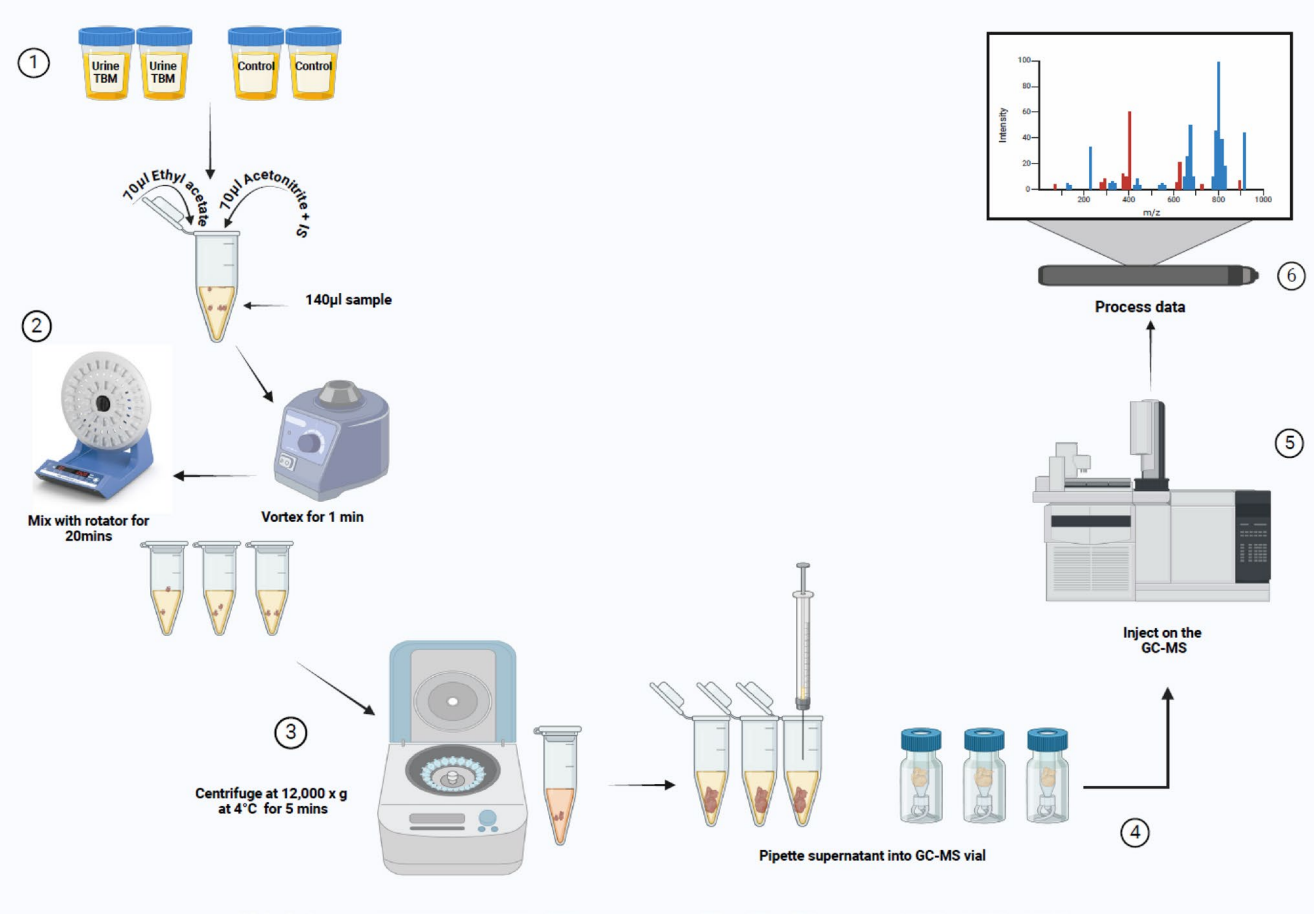
Schematic study design depicting the metabolomics experimental workflow

The Division of Molecular Biology and Human Genetics at Stellenbosch University stored the urine samples in a dedicated freezer at -80 °C. Subsequently, all urine samples designated for metabolomics analysis were sent overnight by courier, frozen and on dry ice, to the Human Metabolomics BSL-3 Laboratory at the North-West University, Potchefstroom campus, where they were aliquoted and stored at -80 °C. A volume of 50 µL was collected from each urine sample and combined into a single tube to form a pooled quality control (QC) urine sample. See Fig. 1 for study design.

### Sample preparation and GC-TOFMS analysis

Before analysis, the samples were defrosted to room temperature in a biological safety cabinet. A 140 µL volume of extraction solvent, containing 70 µL ethyl acetate and 70 µL acetonitrile with an internal standard (IS; 50 ppm concentration of 2E-pentenoic acid), was mixed with 140 µL urine in a ratio of 1:1:2. The material was thoroughly mixed by vortex for 1 min and allowed for proper mixing in a rotator for a further 20 min. Centrifugation at 12,000 x g at 4 °C was performed for 5 min on the 280µL of mixture to facilitate phase separation and sediment any particles and macromolecules. After centrifugation, 120 µL of the top organic phase was transferred to a glass GC vial and sealed. Samples were analysed in a random order, with QC samples inserted at regular intervals, on a GC-TOFMS (Leco Pegasus BT with Agilent 7890 GC).

Prior to analysis, a new liner and septum were installed into the GC-TOFMS to prevent undesired reactions and surface adsorption occurrences. Furthermore, routine maintenance procedures were conducted prior to analysis, encompassing leak detection, tuning, and mass calibration. The samples were analysed in 12 batches, following a randomised approach. A 1 µL volume was injected into the GC-TOFMS using a 1:10 split ratio, with purified helium as a carrier gas, maintained at a constant flow of 1 mL/min. Chromatographic separation was carried out using a Restek Rxi-5MS capillary column (30 m length; 250 μm diameter; 0.25 μm film thickness). Throughout the complete chromatographic procedure, the initial inlet temperature was maintained at 270 °C, the transfer line at 250 °C, and the ion source was set at 200 °C. The initial GC oven protocol began at 50 °C and sustained for 2 min. Subsequently, the temperature was increased in the following manner: 20 °C/min to 120 °C, 16 °C/min to 180 °C, 12 °C/min to 250 °C, and ultimately 8 °C/min to 300 °C, maintained for 1 min. The total duration of each sample analysis was approximately 23 min. Prior to the acquisition of MS data, a 180 s solvent delay was implemented, during which no mass spectra were recorded. Mass spectra were collected across a spectrum of 50–800 m/z at a rate of 20 spectra per second.

### Data pre-processing

Prior to the execution of statistical data analyses, a standardised procedure for the pre-processing of GC-TOFMS metabolomics data was applied. All compounds underwent normalisation utilising the mass spectral total useful signal (MSTUS) by computing the total useful signal for each sample (Chetwynd et al., 2016). Subsequently, variables that exhibited no variation between the groups were excluded, and a data filtering process was executed on each variable to remove those that exhibited more than 50% zero values within each group (Smuts et al., 2013). A quantitative mass merge was then carried out. Thereafter, the coefficient of variation (CV) was evaluated across all quality control (QC) samples, with any variable with a CV > 70% being discarded. All variables with a match of at least 70% with the NIST MS library were labelled as metabolites, and their identities were checked manually afterwards.

### Statistical analysis

All statistical analyses were done using MetaboAnalyst 6.0 (Pang et al., 2024) and Microsoft Excel. Univariate statistical analysis was done when comparing two groups and a one-way ANOVA was done when comparing more than two groups. A Wilcoxon rank-sum test, with multiple testing, was calculated to determine statistical significance (FDR p-value < 0.05). Since our sample size was small, a Hedge’s effect size (instead of a Cohen’s effect size) was calculated to determine practical significance. A small effect (low practical significance) has a g ≤ 0.2, a medium effect (moderate practical significance) has a 0.2 < g ≤ 0.8, and a large effect (high practical significance) has a g > 0.8. All data were log-transformed to allow for visual comparisons between groups (box plots).

## Results

Using an untargeted metabolomics approach, univariate statistical results identified 13 urinary metabolites to be statistically significantly different (FDR p-value < 0.05) when comparing the urine collected from paediatric TBM patients at baseline (T0) and the control group. Six of these 13 compounds had a medium effect size (0.2 < g ≤ 0.8), and seven compounds had a large effect size (g > 0.8). The quantitative univariate statistical measures of these 13 statistically significant compounds are given in Table 1 (see relative abundance of all metabolites in supplementary data). Of these 13 metabolites, three are alkanes and six are VOCs, and the remainder of the results describe these 10 metabolites.

**Table 1:** Statistically significant (FDR p-value < 0.05) compounds in the baseline paediatric TBM patients when compared to the control group

Compound	FDR *p*-value	Hedge’s effect size (g-value)
3-Methoxy-1-pentene	0.014	1.52
4,7-Dimethyl-undecane	0.042	1.39
Pentadecane	0.003	1.30
3-Heptanone	< 0.001	1.27
2-Pyrrolidinone	0.018	1.14
2,6-Dimethyl-undecane	0.042	1.07
Dodecane	0.042	1.05
5,7-Dimethyl-undecane	0.021	0.62
Dichloro-acetic acid, ethyl ester	0.021	0.56
2,5-dimethyl-2-hexene	0.042	0.46
2-Ethyl-trans-2-butenal	0.049	0.44
4,4-Dimethyl-1-pentene	0.042	0.41
2-Butenoic acid, methyl ester	0.018	0.36

Hedge’s effect sizes are also given as g-values, with a medium effect size identified as 0.2 < g ≤ 0.8, and a large effect size identified as g > 0.8

### Alkanes

Pentadecane (a 15-carbon alkane) and three 11-carbon alkanes with two attached methyl groups (5,7-dimethyl-undecane, 4,7-dimethyl-undecane, and 2,6-dimethyl-undecane) were found to be statistically significantly different in the paediatric TBM urine at T0 when compared to the controls. Pentadecane remained significantly increased (FDR *p* < 0.05) throughout the entire treatment duration (6 months of TBM treatment), except at T2 (2 months of TBM treatment) (FDR *p* = 0.083). The Hedge’s effect size g-value of pentadecane remained > 0.8 (large effect) for all TBM treatment points, except at T2, (g = 0.61; medium effect).

4,7-Dimethyl-undecane showed a similar trend to pentadecane– large effect size (g > 0.8) at all TBM treatment points and statistical significance at T3 and T5 when compared to the controls. All four alkanes remained increased and did not return to approximate control levels during TBM treatment. These results suggest that the TBM treatment was insufficient to lower these *M. tb*-related alkanes to control levels.

**Fig. 2:**
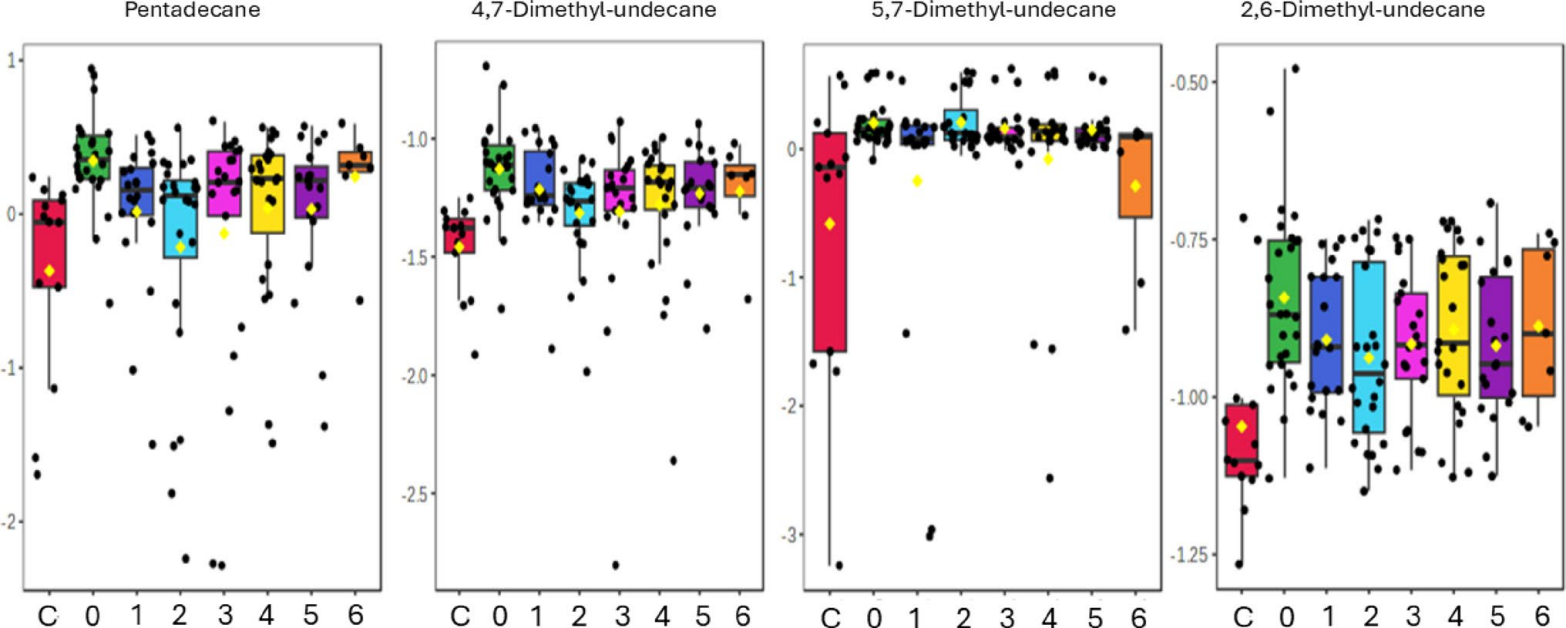
Box plots of the four urinary alkanes identified as showing significant changes across the longitudinal TBM data set. The groups are shown as controls (C; red), baseline TBM (0; green), one month of TB treatment (1; blue), two months of TB treatment (2; light blue), three months of TB treatment (3; pink), four months of TB treatment (4; yellow), five months of TB treatment (5; purple), and six months of TB treatment (6; orange). All data are log-transformed

The other two remaining alkanes (5,7-dimethyl-undecane and 2,6-dimethyl-undecane) also differed statistically significantly in the TBM group at T0 when compared to the controls. However, no statistically significant differences occurred during the TBM treatment; although, they continued to show practical significant differences (medium effect size [0.2 < g ≤ 0.8]) for T1-T6 when compared to the controls. See Fig. 2 for box plots and Table 2 for univariate measures of pentadecane, 5,7-dimethyl-undecane, 4,7-dimethyl-undecane, and 2,6-dimethyl-undecane over the TBM treatment points.

**Table 2:** Four urinary alkanes over TBM treatment points compared to controls

Alkane	Univariate measure	Control vs. TBM treatment points					
		C vs. 1	C vs. 2	C vs. 3	C vs. 4	C vs. 5	C vs. 6
Pentadecane	FDR p-value	0.01	0.083	0.012	0.009	0.007	0.011
	g-value	1.07	0.62	1.04	1.05	1.14	1.48
4,7-Dimethyl-undecane	FDR p-value	0.161	0.083	0.038	0.122	0.031	0.141
	g-value	1.36	0.92	1.01	1.09	1.28	1.57
5,7-Dimethyl-undecane	FDR p-value	0.306	0.083	0.232	0.113	0.299	0.403
	g-value	0.37	0.63	0.43	0.57	0.38	0.40
2,6-Dimethyl-undecane	FDR p-value	0.214	0.39	0.149	0.121	0.614	0.562
	g-value	0.87	0.63	0.81	0.94	0.77	0.95

Statistical significance indicated by FDR p-value < 0.05. Hedge’s effect sizes indicate a medium effect size at 0.2 < g ≤ 0.8 and a large effect size at g > 0.8. The groups are shown as controls (C), one month of TB treatment (1), two months of TB treatment (2), three months of TB treatment (3), four months of TB treatment (4), five months of TB treatment (5), and six months of TB treatment (6)

### Other volatile organic compounds (VOCs)

Three alkenes (3-methoxy-1-pentene; 4,4-dimethyl-1-pentene; 2,5-dimethyl-2-hexene) and an aliphatic ketone (3-heptanone) were also found to be statistically significant. 2,5-Dimethyl-2-hexene and 4,4-dimethyl-1-pentene showed a statistically significant decrease in the TBM baseline samples compared to the controls and remained significantly decreased through the six-month TBM treatment period, with 4,4-dimethyl-1-pentene showing a large effect size (g > 0.8) and 2,5-dimethyl-2-hexene showing a medium effect size (0.2 < g ≤ 0.8). Methoxy-1-pentene was significantly increased at T0, T2-T4, and T6, but decreased (returning close to control levels) at T1 and T5 of TBM treatment. 3-Heptanone remained significantly decreased in the TBM cases throughout treatment, with a large effect size (high practical significance).

**Fig. 3:**
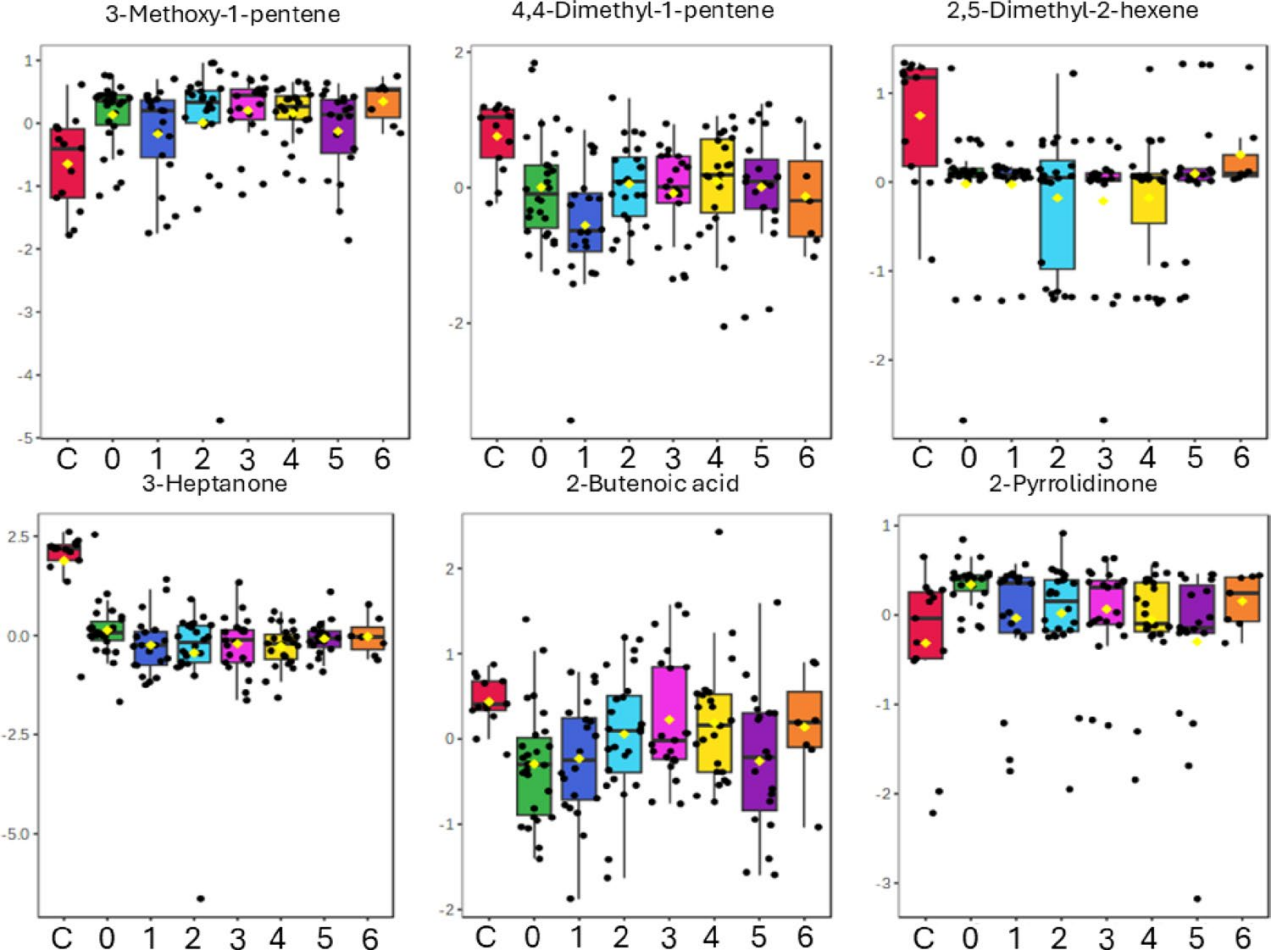
Box plots of six urinary compounds identified as showing significant changes across the longitudinal TBM data set. The groups are shown as controls (C; red), baseline TBM (0; green), one month of TB treatment (1; blue), two months of TB treatment (2; light blue), three months of TB treatment (3; pink), four months of TB treatment (4; yellow), five months of TB treatment (5; purple), and six months of TB treatment (6; orange). All data are log-transformed

2-Butenoic acid methyl ester was significantly decreased and 2-pyrrolidinone was significantly increased at baseline TBM, and 2-butenoic acid methyl ester remained statistically significant at T1. However, for all other time points, these two compounds were not statistically significant but retained a medium effect size. Hence, 2-butenoic acid methyl ester and 2-pyrrolidinone are likely compounds that can be linked to the disease (TBM) because they were statistically significant at TBM baseline and as the disease (but not necessarily the *M. tb* infection) resolved, these compounds also resolved. In particular, 2-pyrrolidinone returned to control levels by T1 of TBM treatment. See Fig. 3 for box plots and Table 3 for the quantitative measures of these six VOCs over the TBM treatment period.

Dichloro-acetic acid, ethyl ester and 2-ethyl-trans-2-butenal are also VOCs (see supplementary data) but no link to biological function could be found in the literature and were thus not considered further in this study.

**Table 3:** Quantitative measures for the other statistically significant VOCs over TBM treatment points compared to controls

Alkane	Univariate measure	Control vs. TBM treatment points					
		C vs. 1	C vs. 2	C vs. 3	C vs. 4	C vs. 5	C vs. 6
3-Methoxy-1-pentene	FDR p-value	0.058	0.006	0.001	0.002	0.064	0.007
	g-value	0.7	1.14	1.59	1.42	0.95	1.64
4,4-Dimethyl-pentene	FDR p-value	< 0,001	0.002	< 0,001	0.002	0.02	0.032
	g-value	2.17	1.51	2.04	1.46	1.29	1.47
2,5-Dimethyl-hexene	FDR p-value	< 0,001	< 0,001	< 0,001	< 0,001	0.028	0.106
	g-value	0.32	0.59	0.35	0.39	0.27	0.32
3-Heptanone	FDR p-value	< 0,001	< 0,001	< 0,001	< 0,001	< 0,001	0.007
	g-value	2.02	2.14	2.05	2.11	1.98	1.54
2-Butenoic acid	FDR p-value	0.009	0.63	0.54	0.581	0.604	0.566
	g-value	0.04	0.14	0.22	0.21	0.22	0.27
2-Pyrrolidinone	FDR p-value	0.241	0.282	0.204	0.581	0.935	0.311
	g-value	0.68	0.49	0.71	0.3	0.38	0.49

Statistical significance indicated by FDR p-value < 0.05. Hedge’s effect sizes indicate a medium effect size at 0.2 < g ≤ 0.8 and a large effect size at g > 0.8. The groups are shown as controls (C), one month of TB treatment (1), two months of TB treatment (2), three months of TB treatment (3), four months of TB treatment (4), five months of TB treatment (5), and six months of TB treatment (6)

## Discussion

In this untargeted GC-TOFMS metabolomics approach, we identified four alkanes and six other VOCs with biological function in the urine of TBM cases at baseline (T0) as being as statistically significant (FDR p-value < 0.05) and/or have medium effect size (0.2 < g ≤ 0.8) or large effect size (g > 0.8). The Hedge’s effect size used in this study provides valuable insights into the practical significance of the variables, going beyond just statistical significance. For more on the value of effect sizes in research, the reader is directed towards Hedges (2008). This discussion will cover the biological significance of these four alkanes and six other VOCs, with a focus on 2-pyrrolidinone, 2-butenoic acid and 3-heptanone.

###  Alkanes

Several species of mycobacteria produce distinctive patterns of VOCs that act as chemical ‘fingerprints’ (Phillips et al., 2007) of the organism and the associated diseases they cause. Alkanes and methylalkanes have been progressively employed by physicians as an innovative approach to the diagnosis of various pathological conditions, circumventing the discomforts associated with invasive methodologies (Cheepsattayakorn & Cheepsattayakorn, 2014). To facilitate the uptake of alkanes, bacteria secrete glycolipid surfactants (Ron & Rosenberg, 2002). The process starts with the oxidation of the terminal methyl group by alkane hydroxylases, leading to the formation of a primary alcohol, which is subsequently oxidised to an aldehyde and finally converted to fatty acids for energy or cell wall biosynthesis (Rojo, 2009). Understanding the adaptation and replication of *M. tb* in the intracellular environment of the host will aid in our understanding of the pathogenesis of TBM. A study by Loots (2014), in an isoniazid-resistant *M. tb* strain, characterised the TetR transcriptional regulator Rv3249c, which regulates a conserved alkane hydroxylase. Rv3249c represses the operon for AlkB, a predicted alkane hydroxylase that enables the utilization of medium- and long-chain (C5-C16) alkanes as carbon sources. It also shows similarity to membrane-bound fatty acid desaturases. Fatty acid desaturation entails the enzymatic removal of hydrogen from a methylene group within an acyl chain (Shanklin & Cahoon, 1998). The findings by Schnappinger et al. (2003) indicate that *M. tb* experiences heightened oxidative stress in the intra-phagosomal environment, leading to increased reliance on fatty acid oxidation for carbon and energy. Loots (2014) also noted that isoniazid-resistant strains with katG mutations likely face greater oxidative stress, prompting enhanced alkane uptake for fatty acid synthesis and energy utilisation. Additionally, alkanes and their derivatives have been detected in human breath as VOCs resulting from oxidative stress (Kneepkens et al., 1994; Phillips et al., 2000). Patients with TBM are known to exhibit increased oxidative stress (Davis et al., 2019). In this current study, we also identified urinary alkenes (2,5-dimethyl-2-hexene; 4,4-dimethyl-1-pentene; 3-methoxy-1-pentene) associated with *M. tb*. Other studies have also reported the presence alkene derivatives in patients with TB (Kolk et al., 2012; Makhubela et al., 2023). Research endeavours should continue to explore the role of VOCs in the diagnosis of TBM. For instance, Phillips et al. (2007) identified a spectrum of VOCs correlated with TB, and 3-heptanone, one of the markers identified in this investigation, has previously been reported as a significant biomarker for breath analysis in the context of TB. Unfortunately, we cannot comment on the possible persistent presence of *M. tb* because the samples used in this study were collected from a retrospective study, and follow-up metadata (e.g. *M. tb* presence/load) at various treatment points and treatment outcomes were not collected (van Elsland et al., 2018).

###  2-Pyrrolidinone

2-Pyrrolidinone is a lactam cyclisation product of the neurotransmitter gamma-aminobutyric acid (GABA) (Hyder et al., 1999). A study (Petroff et al., 1999) also found that antiepileptic drugs increase GABA and its related metabolites homocarnosine and 2-pyrrolidinone. Moreover, 2-pyrrolidinone is seen to induce long-lasting facilitation of hippocampal synaptic transmission by enhancing nicotinic acetylcholine (ACh) receptors in brain responses via a protein kinase C pathway (Miyamoto et al., 2003). AChs contribute to the control of the resting membrane potential, modulation of synaptic transmission, and mediation of fast excitatory transmission (Hogg et al., 2003; Miyamoto et al., 2003). GABA serves as the primary inhibitory neurotransmitter in the human nervous system, and the metabolism of GABA to succinic semialdehyde helps regulate its levels and neurotransmitter activity (Kennedy et al., 2019). The accumulation of GABA, either by the enzymatic inactivity of 4-aminobutyrate aminotransferase (ABAT) or by medical intervention, can result in elevated levels of 2-pyrrolidinone, due to GABA cyclisation (Callery et al., 1978). 2-Pyrrolidinone can also be converted to succinimide through a two-step reaction, and the opening of the hydrolytic ring of cyclic imides such as succinimide can occur through enzymatic and non-enzymatic routes (Kurono et al., 2008; Lerner et al., 2013; Maguire & Dudley, 1978). Inhibition of ABAT activity results in the accumulation of GABA, β-alanine, homocarnosine, and 2-pyrrolidinone (Jaeken et al., 1990; Parviz et al., 2014). Increased levels of GABA lead to symptoms including hypotonia, hyperreflexia, lethargy, refractory seizures, abnormal brain magnetic resonance imaging, and electroencephalogram abnormalities (Dracopoulos et al., 2010; Hussain et al., 2017; Pearl et al., 2009; Schonstedt et al., 2015). Kennedy et al. (2019) compared four patients with GABA-transaminase deficiency, using an untargeted metabolomics approach that compared the biochemical profile in different matrices (plasma, urine, and CSF) for clinical screening of 2-pyrrolidinone and succinimide. Three patients showed elevated levels of 2-pyrrolidinone, succinimide, or its open-ring form (succinamic acid) in plasma, urine, and CSF, and/or homocarnosine in urine and CSF. To elucidate these mechanisms, Kennedy et al. (2019) further indicated that the identification of medications alongside the levels of 2-pyrrolidinone gave insight into the mechanism of the elevation of 2-pyrolidinone as a clinical biomarker. Hence, 2-pyrrolidinone has several links to neurological functions, indicating impairment (shunting) of GABA metabolism in TBM.

### 2-Butenoic acid

2-Butenoic acid is a straight, aliphatic, unsaturated carboxylic acid with a strong apolar nature. 2-Butenoic acid is produced by the oxidation of 2-butanoic acid, coupled with the reduction of NAD^+^ to NADH:

$${\text{Butanoic acid }} + {\text{ NAD}}^{ + } < => {\text{ 2}} - {\text{Butenoate }} + {\text{ NADH }} + {\text{ H}}^{ + }$$

2-Butenoic acid and its derivatives permeate cell membranes through passive diffusion and active transport mechanisms utilising sodium-coupled monocarboxylate transporter-1 or monocarboxylate transporter-1, subsequently undergoing conversion to 2-butenoic acid-CoA, which serves as a principal substrate for the crotonylation of histone lysine residues. However, the comprehensive biological pathways that govern the metabolism of 2-butenoic acid are not yet adequately elucidated (Yang et al., 2023). A segment of the scholarly community posits that 2-butenoic acid, analogous to butyric acid, may function as a metabolic byproduct or an intermediary generated by specific microbial activity in the body; however, empirical substantiation for this hypothesis is currently absent (Fischer et al., 2010). 2-Butenoic acid-CoA, an intermediate metabolite synthesised from fatty acid catabolism, is derived from 2-butenoic acid through the enzymatic action of acyl-CoA synthetase short-chain family (ACSS2) or the oxidative breakdown of amino acids such as lysine, tryptophan, and butyrate (Fang et al., 2021). It has been demonstrated to play a crucial role in a variety of neurological disorders, including neuropathic pain, Alzheimer’s disease, neonatal hypoxic-ischemic encephalopathy, and various neurodevelopmental diseases (Yang et al., 2023). 2-Butenoic acid was also identified as a marker of the presence of poly-β-hydroxybutyrate (PHB), microbial origin (Watt et al., 1991). In the context of aerobic facultative physiology, bacteria exhibit a propensity to amass PHB as a reservoir for carbon and energy. During microbial proliferation and energy production, the tricarboxylic acid (TCA) cycle and the PHB metabolic pathway engage in competition for the substrate acetyl-CoA (Korotkova & Lidstrom, 2001). A study in search of drug targets for *M. tb* (Purohit et al., 2007) showed a truncated citric acid cycle with the glyoxylate shunt, suggesting an option for survival by the pathogen and pathogenesis. Purohit et al. (2007) proposed that precursors to support this pathway could also be generated via enzymatic conversion involving PHB. They supported this claim by using available genome sequence data, which were analysed for possible enzymatic conversions that can generate glyoxylate, acetyl-CoA, and other enolases that can also be useful for various fatty acid transformations. Therefore, 2-butenoic acid is another *M. tb*-related metabolite that needs further investigation.

### 3-Heptanone

3-Heptanone is an aliphatic ketone. The principal metabolite of 3-heptanone has been identified as 2,5-heptanedione, which serves as a neurotoxic analogue of 2,5-hexanedione (O’Donoghue et al., 1984). Notably, 2,5-hexanedione is recognised as the most neurotoxic metabolite arising from the metabolism of n-hexane and methyl n-butyl ketone (MnBK), a neurotoxin that was reported in the literature (Mendell et al., 1974; Spencer et al., 1975). The increased metabolic conversion of 3-heptanone to 2,5-heptanedione may lead to neurotoxic effects (O’Donoghue et al., 1984). 3-Heptanone is also a metabolite of valproic acid (VPA) or 2-propylpentanoic acid, which is classified as branched-chain fatty acid. VPA can exert its effects by inhibiting voltage-gated sodium channels, thereby enhancing the inhibitory actions of GABA (Erhart et al., 2009). The metabolic pathway of VPA includes the conversion to unsaturated compounds via hydrogenation, the generation of hydroxylated metabolites through oxidation, and the formation of carbonyl metabolites through further oxidation. The β-oxidation of VPA produces 3-oxo-VPA, which subsequently undergoes spontaneous decarboxylation to produce 3-heptanone (Erhart et al., 2009; Feriduni et al., 2019). The fact that this particular VOC remained significantly increased (with a very large effect size– high practical significance) in the TBM cases, despite 6 months of TBM treatment, suggests that this VOC is linked to a possible persistent perturbation caused by the *M. tb* infection. Future *M. tb* studies should target this VOC and evaluate its significance.

## Conclusions

To the best of our knowledge, this is the first GC-TOFMS analysis of urinary alkanes and other VOCs in individuals with TBM. This study identified eight compounds possibly related to *M. tb* metabolism: four alkanes (pentadecane, 5,7-dimethyl-undecane, 4,7-dimethyl-undecane, and 2,6-dimethyl-undecane), three alkenes (2,5-dimethyl-2-hexene, 4,4-dimethyl-1-pentene, and 3-methoxy-1-pentene), an aliphatic ketone (3-heptanone), and two additional biological compounds (2-butenoic acid methyl ester and 2-pyrrolidinone). Furthermore, an alternative hypothesis for the persistent perturbation of these urinary VOCs is that they originate from damaged neurons, specifically the myelin sheaths of neurons that contain alkanes, which the *M. tb* bacteria have destroyed (Bourre et al., 1977; Broza et al., 2017; Toshniwal & Zarling, 1992). This suggests that neuronal damage persists throughout TBM treatment and may be permanent.

The limitations of this study include: limited metadata on TBM and control cases, only one sample point for the controls, and the absence of additional groups (e.g., TB or bacterial meningitis) for comparison to ascertain the specificity of these VOCs to TBM. However, this study shows that alkanes and other VOCs in urine are an important consideration in *M. tb*-related diseases and should be further investigated with larger cohorts of *M. tb*-infected individuals and across various biofluids. Therefore, this study improves our understanding of *M. tb* infection but also takes us one step closer towards using urine as a potential non-invasive diagnostic measure for TBM.

## Electronic supplementary material

Below is the link to the electronic supplementary material.

Supplementary Material 1

## Data Availability

The complete data data is given as a supplementary document.

## References

[CR1] Banday, K. M., Pasikanti, K. K., Chan, E. C. Y., Singla, R., Rao, K. V. S., Chauhan, V. S., & Nanda, R. K. (2011). Use of urine volatile organic compounds to discriminate tuberculosis patients from healthy subjects. *Analytical Chemistry*, *83*(14), 5526–5534. 21619052 10.1021/ac200265g

[CR2] Bourre, J. M., Cassagne, C., Larrouquere-Regnier, S., & Darriet, D. (1977). Occurrence of alkanes in brain myelin. Comparison between normal and quaking mouse. *Journal of Neurochemistry*, *29*(4), 645–648. 591942 10.1111/j.1471-4159.1977.tb07781.x

[CR3] Broza, Y. Y., Har-Shai, L., Jeries, R., Cancilla, J. C., Glass-Marmor, L., Lejbkowicz, I., Torrecilla, J. S., Yao, X., Feng, X., Narita, A., & Müllen, K. (2017). Exhaled breath markers for nonimaging and noninvasive measures for detection of multiple sclerosis. *ACS Chemical Neuroscience*, *8*(11), 2402–2413. 28768105 10.1021/acschemneuro.7b00181

[CR4] Callery, P. S., Geelhaar, L. A., & Stogniew, M. (1978). 2-PyrroIidinone—A cyclization product of γ-aminobutyric acid detected in mouse brain. *Biochemical Pharmacology*, *27*, 2061–2063. 718729 10.1016/0006-2952(78)90068-0

[CR5] Cheepsattayakorn, A., & Cheepsattayakorn, R. (2014). Breath tests in diagnosis of pulmonary tuberculosis. *Recent Patents on Biotechnology*, *8*, 172–175. 25185981 10.2174/1872208309666140904115813

[CR6] Chetwynd, A. J., Abdul-Sada, A., Holt, S. G., & Hill, E. M. (2016). Use of a pre-analysis osmolality normalisation method to correct for variable urine concentrations and for improved metabolomic analyses. *Journal of Chromatography A*, *1431*, 103–110. 26755417 10.1016/j.chroma.2015.12.056

[CR7] Churchill, S. A., Harper, J. P., & Churchill, P. F. (1999). Isolation and characterization of a Mycobacterium species capable of degrading three-and four-ring aromatic and aliphatic hydrocarbons. *Applied and Environmental Microbiology*, *65*, 549–552. 9925581 10.1128/aem.65.2.549-552.1999 PMC91060

[CR8] Davis, A. G., Rohlwink, U. K., Proust, A., Figaji, A. A., & Wilkinson, R. J. (2019). The pathogenesis of tuberculous meningitis. *Journal of Leukocyte Biology*, *105*, 267–280. 30645042 10.1002/JLB.MR0318-102R PMC6355360

[CR9] Donald, P., Cotton, M., Hendricks, M., Schaaf, H., de Villiers, J. N., & Willemse, T. (1996). Pediatric meningitis in the Western cape Province of South Africa. *Journal of Tropical Pediatrics*, *42*, 256–261. 8936954 10.1093/tropej/42.5.256

[CR10] Dracopoulos, A., Widjaja, E., Raybaud, C., Westall, C. A., & SneadIII, O. C. (2010). Vigabatrin-associated reversible MRI signal changes in patients with infantile spasms. *Epilepsia*, *51*, 1297–1304. 20384718 10.1111/j.1528-1167.2010.02564.x

[CR11] Dunlap, K., & Perry, J. (1967). Effect of substrate on the fatty acid composition of hydrocarbon-utilizing microorganisms. *Journal of Bacteriology*, *94*, 1919–1923. 6074400 10.1128/jb.94.6.1919-1923.1967 PMC276922

[CR12] Dunlap, K., & Perry, J. (1968). Effect of substrate on the fatty acid composition of hydrocarbon-and ketone-utilizing microorganisms. *Journal of Bacteriology*, *96*, 318–321. 16562157 10.1128/jb.96.2.318-321.1968 PMC252300

[CR13] Erhart, S., Amann, A., Haberlandt, E., Edlinger, G., Schmid, A., Filipiak, W., Schwarz, K., Mochalski, P., Rostasy, K., & Karall, D. (2009). 3-Heptanone as a potential new marker for valproic acid therapy. *Journal of Breath Research*, *3*, 016004. 21383452 10.1088/1752-7155/3/1/016004

[CR14] Fang, Y., Xu, X., Ding, J., Yang, L., Doan, M. T., Karmaus, P. W., Snyder, N. W., Zhao, Y., Li, J. L., & Li, X. (2021). Histone crotonylation promotes mesoendodermal commitment of human embryonicstem cells. *Cell Stem Cell*, *28*(4), 748–763. 10.1016/j.stem.2020.12.009 PMC8026719 33450185

[CR15] Feriduni, B., Barzegar, M., Sadeghvand, S., Shiva, S., Khoubnasabjafari, M., & Jouyban, A. (2019). Determination of valproic acid and 3-heptanone in plasma using air-assisted liquid-liquid Microextraction with the assistance of vortex: Application in the real samples. *Bioimpacts*, *9*, 105–113. 31334042 10.15171/bi.2019.14 PMC6637214

[CR16] Fischer, C. R., Tseng, H. C., Tai, M., Prather, K. L., & Stephanopoulos, G. (2010). Assessment of heterologous butyrate and butanol pathway activity by measurement of intracellular pathway intermediates in Recombinant Escherichia coli. *Applied Microbiology and Biotechnology*, *88*, 265–275. 20625717 10.1007/s00253-010-2749-2 PMC2921503

[CR17] Hedges, L. V. (2008). What are effect sizes and why do we need them? *Child Development Perspectives*, *2*(3), 167–171.

[CR18] Hogg, R., Raggenbass, M., & Bertrand, D. (2003). Nicotinic acetylcholine receptors: From structure to brain function. *Reviews of Physiology Biochemistry and Pharmacology*, *147*, 1–46. 12783266 10.1007/s10254-003-0005-1

[CR19] Hussain, S. A., Tsao, J., Li, M., Schwarz, M. D., Zhou, R., Wu, J. Y., Salamon, N., & Sankar, R. (2017). Risk of vigabatrin-associated brain abnormalities on MRI in the treatment of infantile spasms is dose‐dependent. *Epilepsia*, *58*, 674–682. 28230253 10.1111/epi.13712

[CR20] Hyder, F., Petroff, O. A., Mattson, R. H., & Rothman, D. L. (1999). Localized 1H NMR measurements of 2-pyrrolidinone in human brain in vivo. *Magnetic Resonance in Medicine: an Official Journal of the International Society for Magnetic Resonance in Medicine*, *41*, 889–896. 10.1002/(sici)1522-2594(199905)41:5<889::aid-mrm6>3.0.co;2-r 10332870

[CR21] Jaeken, J., Casaer, P., Haegele, K., & Schechter, P. (1990). Normal and abnormal central nervous system GABA metabolism in childhood. *Journal of Inherited Metabolic Disease*, *13*, 793–801. 2079831 10.1007/BF01800202

[CR22] Kennedy, A. D., Pappan, K. L., Donti, T., Delgado, M. R., Shinawi, M., Pearson, T. S., Lalani, S. R., Craigen, W. J., Sutton, V. R., & Evans, A. M. (2019). 2-Pyrrolidinone and succinimide as clinical screening biomarkers for GABA-transaminase deficiency: Anti-seizure medications impact accurate diagnosis. *Frontiers in Neuroscience*, *13*, 394. 31133775 10.3389/fnins.2019.00394 PMC6517487

[CR23] Kneepkens, C. F., Lepage, G., & Roy, C. C. (1994). The potential of the hydrocarbon breath test as a measure of lipid peroxidation. *Free Radical Biology and Medicine*, *17*, 127–160. 7959173 10.1016/0891-5849(94)90110-4

[CR24] Kolk, A., Van Berkel, J., Claassens, M., Walters, E., Kuijper, S., Dallinga, J., & Van Schooten, F. (2012). Breath analysis as a potential diagnostic tool for tuberculosis. *The International Journal of Tuberculosis and Lung Disease*, *16*, 777–782. 22507235 10.5588/ijtld.11.0576

[CR25] Korotkova, N., & Lidstrom, M. E. (2001). Connection between poly-β-hydroxybutyrate biosynthesis and growth on C1 and C2 compounds in the Methylotroph Methylobacterium extorquens AM1. *Journal of Bacteriology*, *183*, 1038–1046. 11208803 10.1128/JB.183.3.1038-1046.2001 PMC94972

[CR26] Kurono, M., Itogawa, A., Noguchi, H., & Sanjoba, M. (2008). Stability and hydrolysis kinetics of spirosuccinimide type inhibitors of aldose reductase in aqueous solution and retardation of their hydrolysis by the target enzyme. *Journal of Pharmaceutical Sciences*, *97*, 1468–1483. 17722083 10.1002/jps.21049

[CR27] Lerner, E., Hilzenrat, G., Amir, D., Tauber, E., Garini, Y., & Haas, E. (2013). Preparation of homogeneous samples of double-labelled protein suitable for single-molecule FRET measurements. *Analytical and Bioanalytical Chemistry*, *405*, 5983–5991. 23649926 10.1007/s00216-013-7002-2

[CR28] Lim, S. H., Martino, R., Anikst, V., Xu, Z., Mix, S., Benjamin, R., Schub, H., Eiden, M., Rhodes, P. A., & Banaei, N. (2016). Rapid diagnosis of tuberculosis from analysis of urine volatile organic compounds. *ACS Sensors*, *1*(7), 852–856. 29057329 10.1021/acssensors.6b00309 PMC5648341

[CR29] Loots, D. T. (2014). An altered mycobacterium tuberculosis metabolome induced by *KatG* mutations resulting in Isoniazid resistance. *Antimicrobial Agents and Chemotherapy*, *58*, 2144–2149. 24468786 10.1128/AAC.02344-13 PMC4023710

[CR30] Maguire, J. H., & Dudley, K. H. (1978). Dihydropyrimidinase. Metabolism of some Cyclic Imides of different ring size. *Drug Metabolism and Disposition*, *6*, 140–145. 26528

[CR31] Makhubela, P. C. K., Rohwer, E. R., & Naudé, Y. (2023). Detection of tuberculosis-associated compounds from human skin by GCxGC-TOFMS. *Journal of Chromatography B*, *1231*, 123937. 10.1016/j.jchromb.2023.123937 37995549

[CR32] Mandalakas, A. M., Hesseling, A. C., Kay, A., Du Preez, K., Martinez, L., Ronge, L., DiNardo, A., Lange, C., & Kirchner, H. L. (2021). Tuberculosis prevention in children: A prospective community-based study in South Africa. *European Respiratory Journal*, *57*, 2003028. 33122339 10.1183/13993003.03028-2020 PMC8060782

[CR33] Marais, S., Thwaites, G., Schoeman, J. F., Török, M. E., Misra, U. K., Prasad, K., Donald, P. R., Wilkinson, R. J., & Marais, B. J. (2010). Tuberculous meningitis: A uniform case definition for use in clinical research. *The Lancet Infectious Diseases*, *10*, 803–812. 20822958 10.1016/S1473-3099(10)70138-9

[CR34] Mendell, J. R., Saida, K., Ganansia, M., Jackson, D., Weiss, H., Gardier, R., Chrisman, C., Allen, N., Couri, D., & O’Neill, J. (1974). Toxic polyneuropathy produced by Methyl n-butyl ketone. *Science*, *185*, 787–789. 4367263 10.1126/science.185.4153.787

[CR35] Miyamoto, H., Yaguchi, T., Ohta, K., Nagai, K., Nagata, T., Yamamoto, S., & Nishizaki, T. (2003). 2-pyrrolidinone induces a long-lasting facilitation of hippocampal synaptic transmission by enhancing α7 ach receptor responses via a PKC pathway. *Molecular Brain Research*, *117*, 91–96. 14499485 10.1016/s0169-328x(03)00281-x

[CR36] O’Donoghue, J. L., Krasavage, W. J., DiVincenzo, G. D., & Katz, G. V. (1984). Further studies on ketone neurotoxicity and interactions. *Toxicology and Applied Pharmacology*, *72*, 201–209. 6546457 10.1016/0041-008x(84)90304-1

[CR37] Pang, Z., Lu, Y., Zhou, G., Hui, F., Xu, L., Viau, C., Spigelman, A. F., MacDonald, P. E., Wishart, D. S., Li, S., & Xia, J. (2024). MetaboAnalyst 6.0: Towards a unified platform for metabolomics data processing, analysis and interpretation. *Nucleic Acids Research*, *52*, W398–W406. 38587201 10.1093/nar/gkae253 PMC11223798

[CR38] Parviz, M., Vogel, K., Gibson, K. M., & Pearl, P. L. (2014). Disorders of GABA metabolism: SSADH and GABA-transaminase deficiencies. *Journal of Pediatric Epilepsy*, *3*, 217–227. 25485164 10.3233/PEP-14097 PMC4256671

[CR39] Pearl, P. L., Vezina, L. G., Saneto, R. P., McCarter, R., Molloy-Wells, E., Heffron, A., Trzcinski, S., McClintock, W. M., Conry, J. A., & Elling, N. J. (2009). Cerebral MRI abnormalities associated with Vigabatrin therapy. *Epilepsia*, *50*, 184–194. 18783433 10.1111/j.1528-1167.2008.01728.x

[CR40] Petroff, O. A., Hyder, F., Collins, T., Mattson, R. H., & Rothman, D. L. (1999). Acute effects of Vigabatrin on brain GABA and Homocarnosine in patients with complex partial seizures. *Epilepsia*, *40*, 958–964. 10403220 10.1111/j.1528-1157.1999.tb00803.x

[CR41] Phillips, M., Basa-Dalay, V., Bothamley, G., Cataneo, R. N., Lam, P. K., Natividad, M. P. R., Schmitt, P., & Wai, J. (2010). Breath biomarkers of active pulmonary tuberculosis. *Tuberculosis*, *90*, 145–151. 20189456 10.1016/j.tube.2010.01.003

[CR42] Phillips, M., Cataneo, R. N., Condos, R., Erickson, G. A. R., Greenberg, J., La Bombardi, V., Munawar, M. I., & Tietje, O. (2007). Volatile biomarkers of pulmonary tuberculosis in the breath. *Tuberculosis*, *87*, 44–52. 16635588 10.1016/j.tube.2006.03.004

[CR43] Phillips, M., Cataneo, R. N., Greenberg, J., Gunawardena, R., Naidu, A., & Rahbari-Oskoui, F. (2000). Effect of age on the breath methylated alkane contour, a display of apparent new markers of oxidative stress. *Journal of Laboratory and Clinical Medicine*, *136*, 243–249. 10985503 10.1067/mlc.2000.108943

[CR44] Purohit, H. J., Cheema, S., Lal, S., Raut, C. P., & Kalia, V. C. (2007). In search of drug targets for Mycobacterium tuberculosis. *Infectious Disorders-Drug Targets (Formerly Current Drug Targets-Infectious Disorders)*, *7*, 245–250. 10.2174/187152607782110068 17897060

[CR45] Rojo, F. (2009). Degradation of alkanes by bacteria. *Environmental Microbiology*, *11*, 2477–2490. 19807712 10.1111/j.1462-2920.2009.01948.x

[CR46] Ron, E. Z., & Rosenberg, E. (2002). Biosurfactants and oil bioremediation. *Current Opinion in Biotechnology*, *13*, 249–252. 12180101 10.1016/s0958-1669(02)00316-6

[CR47] Schnappinger, D., Ehrt, S., Voskuil, M. I., Liu, Y., Mangan, J. A., Monahan, I. M., Dolganov, G., Efron, B., Butcher, P. D., & Nathan, C. (2003). Transcriptional adaptation of Mycobacterium tuberculosis within macrophages: Insights into the phagosomal environment. *The Journal of Experimental Medicine*, *198*, 693–704. 12953091 10.1084/jem.20030846 PMC2194186

[CR48] Schonstedt, V., Stecher, X., Venegas, V., & Silva, C. (2015). Vigabatrin-induced MRI changes associated with extrapyramidal symptoms in a child with infantile spasms. *The Neuroradiology Journal*, *28*, 515–518. 26306928 10.1177/1971400915598082 PMC4757222

[CR49] Shanklin, J., & Cahoon, E. B. (1998). Desaturation and related modifications of fatty acids. *Annual Review of Plant Biology*, *49*, 611–641. 10.1146/annurev.arplant.49.1.611 15012248

[CR50] Smits, T. H., Balada, S. B., Witholt, B., & Van Beilen, J. B. (2002). Functional analysis of alkane hydroxylases from gram-negative and gram-positive bacteria. *Journal of Bacteriology*, *184*, 1733–1742. 11872725 10.1128/JB.184.6.1733-1742.2002 PMC134907

[CR51] Smuts, I., van der Westhuizen, F. H., Louw, R., Mienie, L. J., Engelke, U. F. H., Wevers, R. A., Mason, S., Koekemoer, G., & Reinecke, C. J. (2013). Disclosure of a putative biosignature for respiratory chain disorders through a metabolomics approach. *Metabolomics*, *9*, 379–391.

[CR52] Spencer, P. S., Schaumburg, H. H., Raleigh, R. L., & Terhaar, C. J. (1975). Nervous system degeneration produced by the industrial solvent Methyl n-butyl ketone. *Archives of Neurology*, *32*, 219–222. 1124984 10.1001/archneur.1975.00490460035002

[CR53] Stokas, H., Rhodes, H. L., Simmons, M. B., Zhang, R., Wright, C. C., & Purdy, G. E. (2022). M. tuberculosis alkx encoded by rv3249c regulates a conserved alkane hydroxylase system that is important for replication in macrophages and biofilm formation. *Microbiology Spectrum*, *10*, e01969–e01922. 35938806 10.1128/spectrum.01969-22 PMC9430723

[CR54] Toshniwal, P. K., & Zarling, E. J. (1992). Evidence for increased lipid peroxidation in multiple sclerosis. *Neurochemical Research*, *17*, 205–207. 1538834 10.1007/BF00966801

[CR55] Van Beilen, J. B., Smits, T. H., Whyte, L. G., Schorcht, S., Röthlisberger, M., Plaggemeier, T., Engesser, K. H., & Witholt, B. (2002). Alkane hydroxylase homologues in Gram-positive strains. *Environmental Microbiology*, *4*, 676–682. 12460275 10.1046/j.1462-2920.2002.00355.x

[CR56] van Elsland, S. L., Peters, R. P., Kok, M. O., van Toorn, R., Springer, P., Cotton, M. F., Grobbelaar, C. J., Aarnoutse, R., & van Furth, A. M. (2018). A treatment-support intervention evaluated in South African paediatric populations with HIV infection or tuberculous meningitis. *Tropical Medicine & International Health*, *23*, 1129–1140. 30075490 10.1111/tmi.13134

[CR57] van Toorn, R., & Solomons, R. (2014). Update on the diagnosis and management of tuberculous meningitis in children. *Seminars in Pediatric Neurology*, *21*(1), 12–18. 24655399 10.1016/j.spen.2014.01.006

[CR58] Van Toorn, R., Springer, P., Laubscher, J., & Schoeman, J. (2012). Value of different staging systems for predicting neurological outcome in childhood tuberculous meningitis. *The International Journal of Tuberculosis and Lung Disease*, *16*, 628–632. 22410643 10.5588/ijtld.11.0648

[CR59] Watt, B. E., Morgan, S. L., & Fox, A. (1991). 2-Butenoic acid, a chemical marker for poly-β-hydroxybutyrate identified by pyrolysis—gas chromatography/mass spectrometry in analyses of whole microbial cells. *Journal of Analytical and Applied Pyrolysis*, *19*, 237–249.

[CR60] Wentzel, A., Ellingsen, T. E., Kotlar, H. K., Zotchev, S. B., & Throne-Holst, M. (2007). Bacterial metabolism of long-chain n-alkanes. *Applied Microbiology and Biotechnology*, *76*, 1209–1221. 17673997 10.1007/s00253-007-1119-1

[CR61] Yang, P., Qin, Y., Zeng, L., He, Y., Xie, Y., Cheng, X., Huang, W., & Cao, L. (2023). Crotonylation and disease: Current progress and future perspectives. *Biomedicine & Pharmacotherapy*, *165*, 115108. 37392654 10.1016/j.biopha.2023.115108

